# Study of the Impact on Zygomatic Bone Using Numerical Simulation

**DOI:** 10.3390/biomimetics9110696

**Published:** 2024-11-14

**Authors:** Gonzalo Ruiz-de-León, María Baus-Domínguez, Maribel González-Martín, Aida Gutiérrez-Corrales, Eusebio Torres-Carranza, Álvaro-José Martínez-González, Daniel Torres-Lagares, José-Manuel López-Millan, Jesús Ambrosiani-Fernández

**Affiliations:** 1 Departament of Dentistry, Faculty of Dentistry, University of Sevilla, C/Avicena S/N, 41009 Seville, Spain; migm@us.es (M.G.-M.); agcorrales@us.es (A.G.-C.); danieltl@us.es (D.T.-L.); 2 Department of Oral and Maxillofacial Surgery, Virgen del Rocio University Hospital, 41013 Seville, Spain; drtorres@clinicatorrescarranza.es; 3 ICEMM S.L.U., Engineering Specialized in FEM and CFD Simulation, C/las Fábricas, 28923 Alcorcón, Spain; alvaro.martinez@icemm.es; 4 Department of Surgery, Faculty of Medicine, University of Seville, 41009 Seville, Spain; joselopezmillan@us.es; 5 Department of Anatomy and Embryology, Faculty of Medicine, University of Seville, 41009 Seville, Spain; ambrosiani@us.es

**Keywords:** impact analysis, zygomatic bone, finite-element method, zygomatic implants

## Abstract

The zygomatic bone, a fundamental structure in facial anatomy, is exposed to fractures in impact situations, such as traffic accidents or contact sports. The installation of zygomatic implants can also alter the distribution of forces in this region, increasing the risk of fractures. To evaluate this situation, the first step is to develop a complex anatomical model from the stomatognathic point of view so that simulations in this sense can be validated. This study uses numerical simulation using a finite-element method (FEM) to analyze the behavior of the zygomatic bone under impacts of different velocities, offering a more realistic approach than previous studies by including the mandible, cervical spine, and masticatory muscles. Methods: An FEM model was developed based on 3D scans of actual bones, and simulations were performed using Abaqus Explicit 2023 software (Dassault Systemes, Vélizy-Villacoublay, France). The impact was evaluated using a steel cylinder (200 mm length, 40 mm diameter, 2 kg weight) impacted at speeds of 5, 10, 15, and 20 km/h. Zygomatic, maxillary, and mandibular bone properties were based on dynamic stiffness parameters, and bone damage was analyzed using ductile fracture and fracture energy criteria. Results: The results show that at impact velocities of 15 and 20 km/h, the zygomatic bone suffered crush fractures, with impact forces up to 400 kg. At 10 km/h, a combination of crushing and bending was observed, while at 5 km/h, only local damage without complete fracture was detected. The maximum stresses were concentrated at the zygoma–jaw junction, with values above 100 MPa at some critical points. Conclusion: The FEM model developed offers a detailed representation of the mechanical behavior, integrating the main structures of the stomatognathic apparatus of the zygomatic bone under impact, providing valuable information to, for example, advance injury prevention and zygomatic implant design. Higher impact velocities result in severe fractures, underscoring the need for protective measures in clinical and sports settings.

## 1. Introduction

The use of numerical simulations using the finite-element method (FEM) has revolutionized biomechanics, making it possible to study with great precision how bones respond to different loading conditions. In particular, the zygomatic bone, also known as the cheekbone, plays a vital role in the structure of the facial mass, forming part of the ocular orbit and acting as a bridge between the maxilla and the temporal bone [1,2]. This bone is involved in essential functions, such as mastication, as it serves as an insertion point for powerful muscles such as the masseter, which transmit considerable forces to the face during orofacial activity [1,2].

Anatomically, the zygomatic bone has a quadrangular shape and is connected to the rest of the skull through necessary sutures: the zygomatic–maxillary, zygomatic–temporal, and zygomatic–frontal sutures. These sutures facilitate the transmission of forces between the zygoma and adjacent structures [1,2] but also represent areas of potential weakness during impacts. The zygomatic bone is irrigated at the vascular level by branches of the facial artery and the superficial temporal artery. This makes it a well-vascularized region but also vulnerable in case of fracture or trauma. In terms of innervation, it receives branches of the zygomatic nerve, a branch of the maxillary nerve (V2), which means that fractures in this region can compromise facial sensitivity and muscle function [1,2].

Due to its prominence and direct exposure, the zygomatic bone is most susceptible to fractures in traffic accidents, falls, or contact sports. These fractures, in addition to compromising facial esthetics, can affect masticatory function and cause neurological and vascular complications if not adequately treated [3,4].

Using finite elements to simulate bone fractures is not a recent approach [5]. According to Mischinski and Ural (2013) [6], fracture propagation in human cortical bone is influenced by several microstructural variables, such as the strength and fracture toughness of cement lines and osteones. The internal structure, which includes the trabecular bone of the zygoma, is designed to withstand these forces. The trabeculae of the bone are oriented to resist the stresses generated during orofacial activities, evidencing a direct relationship between the internal morphology of the zygomatic bone and its function in mastication and other oral activities [7].

The present study focuses on the simulation of the mechanical behavior of the zygomatic bone under different impact conditions, using an FEM model that incorporates not only the zygomatic bone but also adjacent structures, such as the mandible, cervical spine, and masticatory muscles, dynamically adjusted to accurate tests [8]. Unlike previous studies that treat the zygomatic bone rigidly, this work presents a more realistic setup that simulates the flexibility and dynamics between the structures involved in the entire stomatognathic field. This approach is fundamental to better understanding how fractures occur and how external factors, such as the insertion of zygomatic implants or dental prostheses, influence them.

## 2. Materials and Methods

### 2.1. Description of the Simulation Model and Finite Element Analysis

The starting FEM (finite element simulation and analysis) model is the Total Human Model for Safety (THUMS) (AM50 Occupant Model Version 6.1 (2021)), a finite-element model of the human body created in collaboration between Toyota Motor Corporation and Toyota Central R&D Labs, Inc. THUMS has hundreds of materials to accurately model the behavior of bone, tendons, muscles, and other soft tissues, both in elastic and plastic regimes and failure due to breakage. This model, however, does not present a sufficient level of detail for the evaluation of the behavior of the maxillary bones; so, these bones are replaced in the source model by others derived from real geometry obtained via CBCT (Orthopos SL 3d, Denstply International Inc., Charlotte, NC, USA).

The definition of the materials used for the mandible and maxillary bones was based on Misch’s classification [9,10], where a type II bone with a cortical thickness varying between 1.5 mm and 2.0 mm was chosen as the configuration for the mandible, maxilla, and zygomatic bones (Table 1).

### 2.2. Description of Fracture and Damage Analysis

For fracture and damage analysis, a ductile fracture criterion based on a maximum deformation of 3% for cortical bone has been chosen. This criterion is based on the THUMS model together with that described by Burstein et al. (1976) [11] and McCalden et al. (1993) [12].

The maximum deformation for trabecular bone is greater than 20%; however, for the simulations, it has been conservatively limited to twice the deformation due to cortical bone breakage.

Table 2 shows the parameters that allow for the initiation and propagation of damage according to the ductile rupture criterion to be defined.

A term based on fracture energy has been chosen as a damage evolution criterion; its value is G = 1.54 [mJ/mm^2^].

The strain rate dependence has been performed using the Cowper–Symonds law, which allows for calculating the parameter *R* as a function of the strain rate according to the following expression:

$${\dot{\underline{\varepsilon }}}^{pl}=D{\left(R-1\right)}^{n}$$

where the following parameters have been defined according to THUMS:

*D* = 360.7
*n* = 4.61

**Figure 1:**
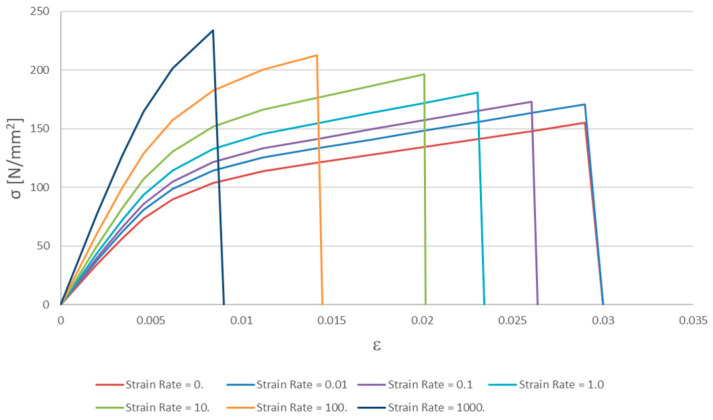
Cortical bone. True stress–strain curves.

The stress–strain curves for cortical bone as a function of strain rate are plotted in Figure 1.

**Figure 2:**
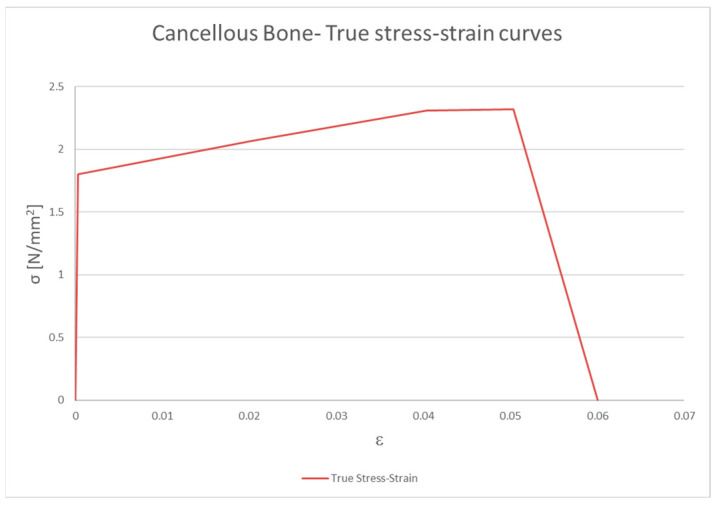
Cancellous bone. True stress–strain curves.

Trabecular bone, which is subjected to secondary breakage after cortical bone breakage, has been defined independently of the strain rate because this property has a low impact on bone breakage. The curve defined for trabecular bone is shown in Figure 2.

### 2.3. Description of the Impact

**Figure 3:**
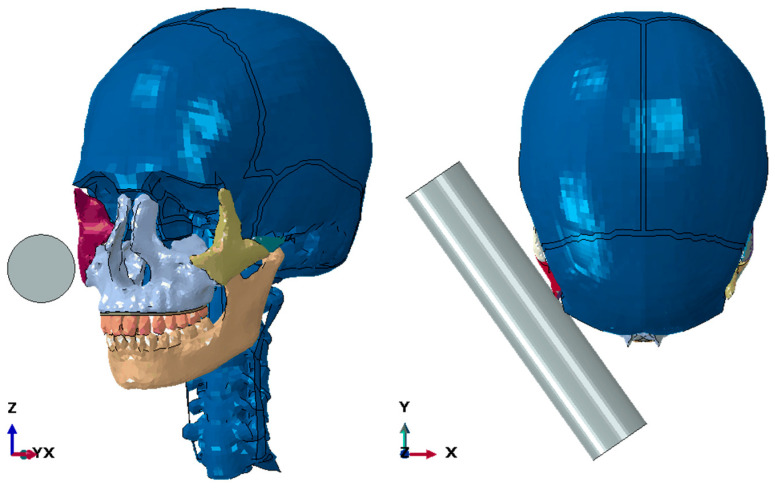
Impactor position.

A steel cylinder of length 200 mm, diameter 40 mm, and weight 2 kg was used as an impactor (Figure 3).

Impacts at 5, 10, 15, and 20 km/h were evaluated.

### 2.4. Finite-Element Model Analysis

All analyses have been performed using the finite-element method and the commercial software Abaqus Explicit 2023 (Dassault Systemes, Vélizy-Villacoublay, France).

**Figure 4:**
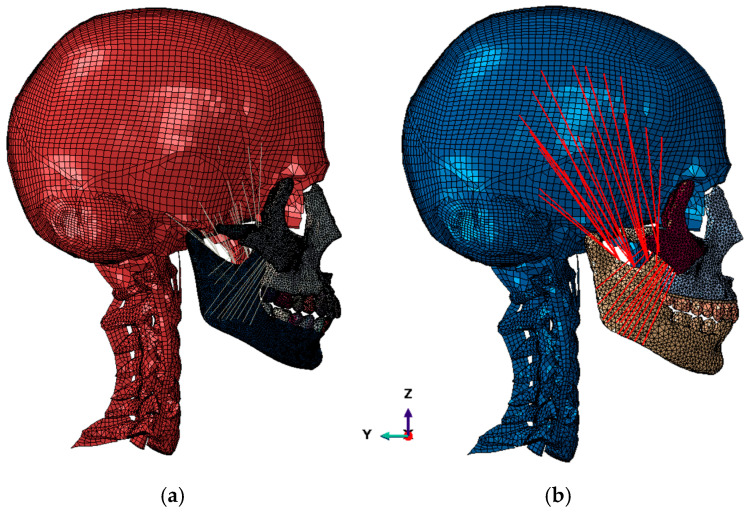
FEM model: (**a**) FEM model with detailed dentition; (**b**) FEM model with simplified dentition. The red lines refer to the masticatory muscles represented and integrated in the model.

Two different finite-element models were created: one that included the teeth and tooth sockets and another more simplified model in which the lower teeth were integrated into the mandible and the upper teeth were placed on a prosthetic base rigidly attached to the maxillary bone (Figure 4). As the impact zone is away from the teeth, the way the teeth were modeled does not affect the results obtained.

Conversely, defining this second model allows us to have larger element sizes and incorporate future prosthetic configurations in impact studies.

Both models cover the head and neck up to the C7 cervical vertebra, and all soft tissues except skin, eyes, and ears have been maintained.

**Figure 5:**
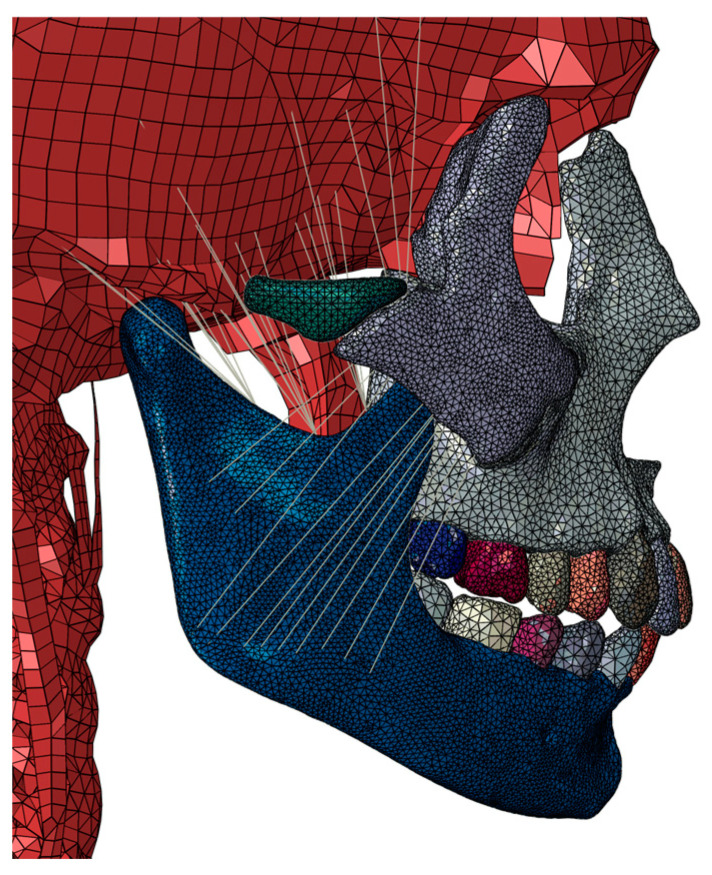
FEM model of bite analysis with intact dentition. Detail—lateral view. The grey lines refer to the masticatory muscles represented and integrated in the model.

Muscles and tendons are modeled using CONN3D2 connector-type elements, where interpolation elements make the connection between the connector and the bone without stiffness to distribute the load of the connector over an area of influence. The type of element used to model the maxilla, zygomatic, mandible, and teeth is C3D10 s-order tetrahedra, with an average mesh size of 1–1.5 mm [13]. The rest of the parts of the model are those derived from the original model according to THUMS and what is described by Azcarate-Velázquez et al. (2019) [13] with first-order hexahedral elements C3D8 for the skull and first-order tetrahedra C3D4 for vertebrae and parts of complex geometry. The total number of elements is 1.68 million elements (Figure 5).

As boundary conditions, all degrees of freedom have been restricted to the nodes that form the base of the C7 vertebra.

**Figure 6:**
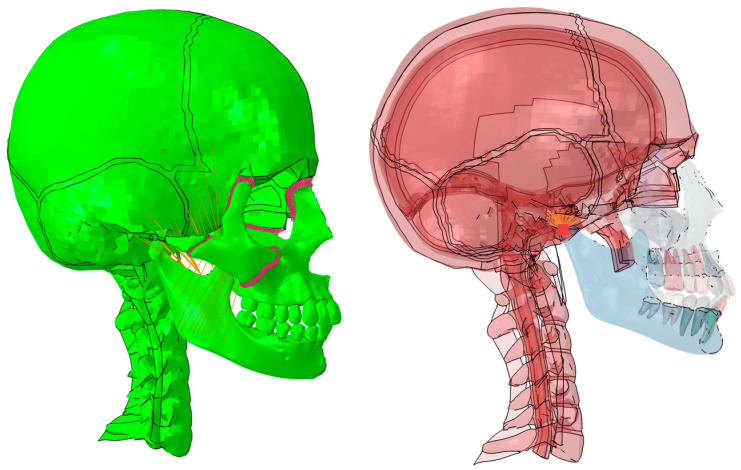
FEM model of bite analysis with intact dentition. Temporomandibular joint: (**Left**), an anatomical model with solid bone. (**Right**), an anatomical model with a transparent bone to better observe the internal structures.

The connection between the maxilla, zygomatic bone, and the skull was made using tie-type kinematic restraints, which rigidly join two surfaces. Meanwhile, the connection between the mandible and the skull has been made using a JOIN connector with free degrees of freedom to rotate to simulate the temporomandibular joint (Figure 6).

### 2.5. Contact Interaction Between the Impactor and Zygomatic Bone

The interaction between bone and impactor is not direct, as the skin is considered as intermediate soft tissue, which acts as a transmitter of contact forces. The study by Huempfner-Hierl et al. (2015) [14] analyzes the effect of including skin in impact analyses on the zygomatic bone. The results indicate that no significant alterations are observed in the behavior of the bone upon impact, although there is a temporary delay in reaching the maximum impact force. In that investigation, skin thicknesses of approximately 9 mm were used in the zygomatic bone region [14]. However, later studies on skin thickness in that same area suggest values of 1.5 mm, with tolerances of ±0.5 mm [15,16].

The skin Is not explicitly Incorporated In our study model, but Its effect has been considered through a contact formulation. For this purpose, an exponential penetration contact law with clearance, c_0_ = 1.5 mm, has been implemented to assess the average skin thickness of 1.5 mm in the zygomatic bone area, and p_0_ = 100 N/mm^2^.

**Figure 7:**
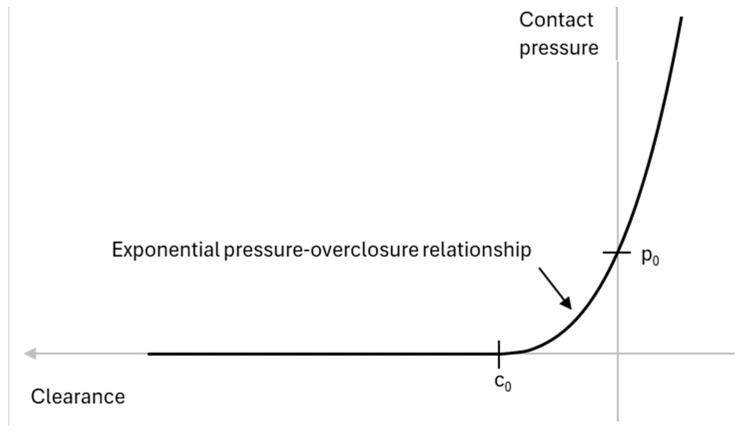
Impactor–bone contact law.

Figure 7 shows the contact law used.

A friction coefficient 0.2 between the impactor and bone has also been considered.

The interaction between the skull bones has been defined as “hard contact”, with a friction coefficient of 0.2.

#### 2.5.1. Impact Simulation Parameters

The impact analysis used the explicit finite element code Abaqus Explicit (Dassault Systemes, Vélizy-Villacoublay, France).

The simulation time was 0.02 s, with a minimum time step of 1 × 10^−7^. This time-step has been controlled by introducing a mass scaling factor for those elements that require a stable time lower than the defined one. The elements affected by mass scaling represent less than 1% of the model mass, so its use is appropriate to reduce computational time.

#### 2.5.2. Dynamic Model Fitting

**Figure 8:**
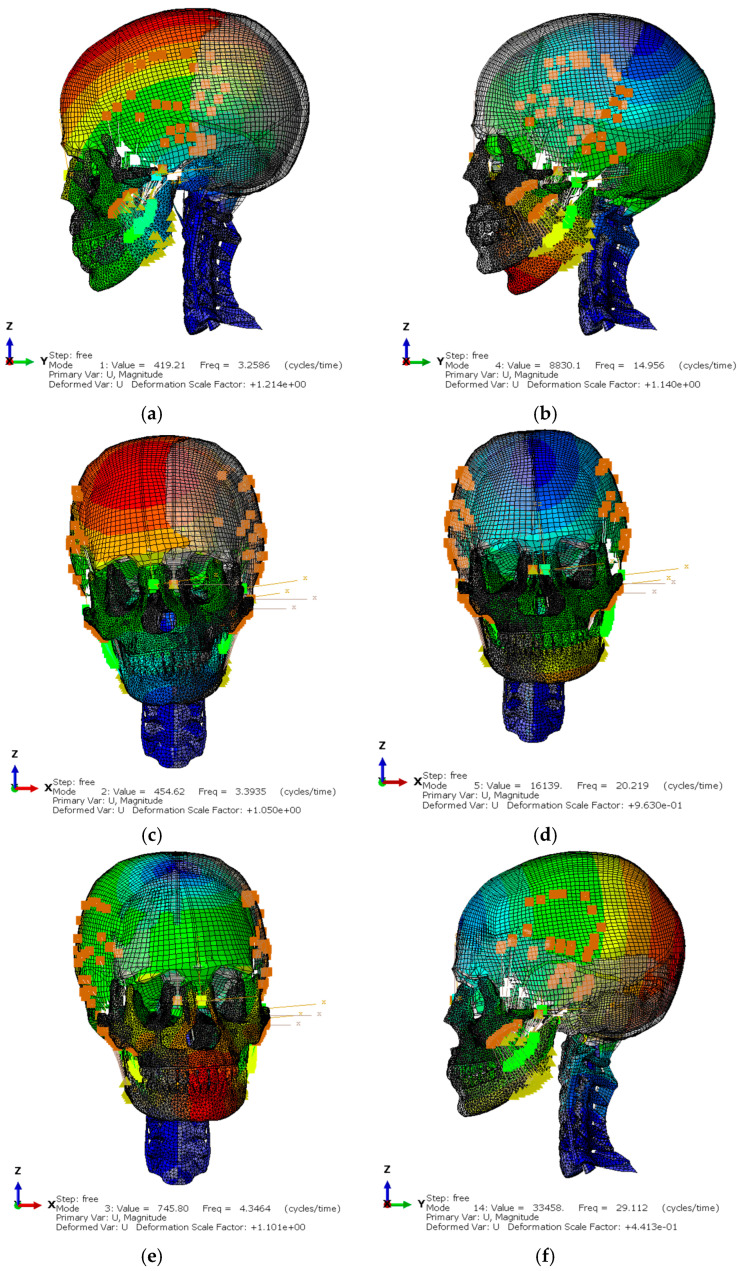
Dynamic FEM model: (**a**) frontal flexion. 3.26 Hz; (**b**) second frontal flexion. 14.96 Hz; (**c**) second lateral flexion. 3.39 Hz; (**d**) lateral flexion. 20.22 Hz; (**e**) torsion. 4.35 Hz; (**f**) axial flexion. 29.11 Hz.

The FEM model has been fitted and validated by comparison of the principal modes of vibration [8] at front bending, second front bending, lateral bending, second lateral bending, torsion, and axial bending (Figure 8).

## 3. Results

The results below refer to two evaluation parameters: the impact force and the damage caused to the zygomatic bone.

### 3.1. Impact Forces

**Figure 9:**
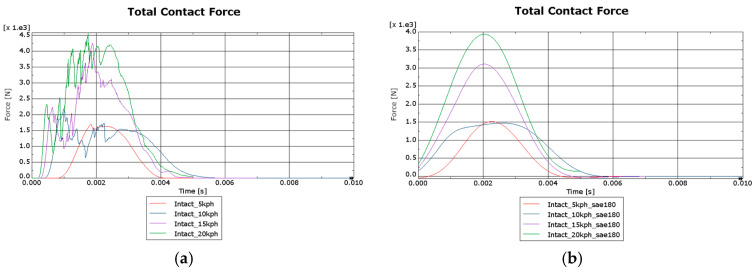
Total contact force: (**a**) unfiltered impact forces; (**b**) impact forces filtered with SAE180 standard in Abaqus CAE.

Referring to the impact force, the following curves force [N]–time [s] represented in Figure 9 have been obtained.

### 3.2. Evolution of the Bone Fracture

**Figure 10:**
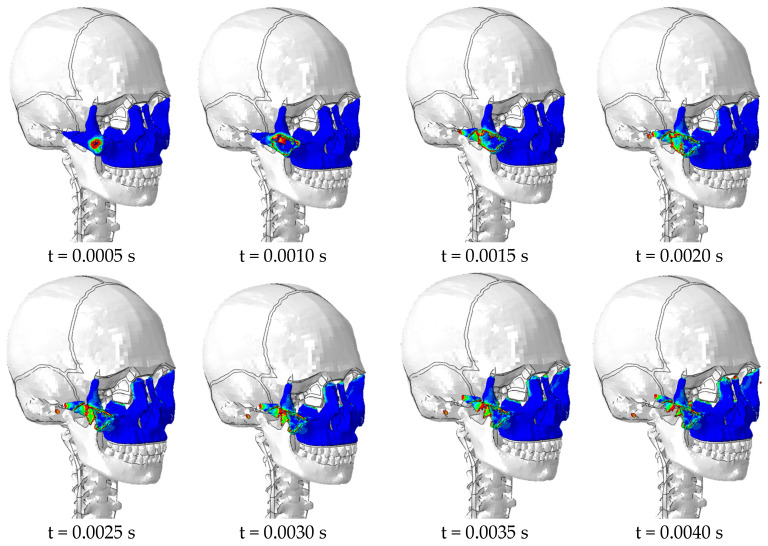
Evolution of zygomatic bone damage for 20 km/h impact. The entire middle third of the face is highlighted in blue. The red field indicates the initiation of bone damage.

The evolution of bone fracture with impact time for an impact velocity of 20 km/h, where the evaluation parameter DUCTCRIT gives the value of bone damage initiation for values greater than or equal to 1.0 (red field), is plotted in Figure 10.

### 3.3. Fracture Lines

**Figure 11:**
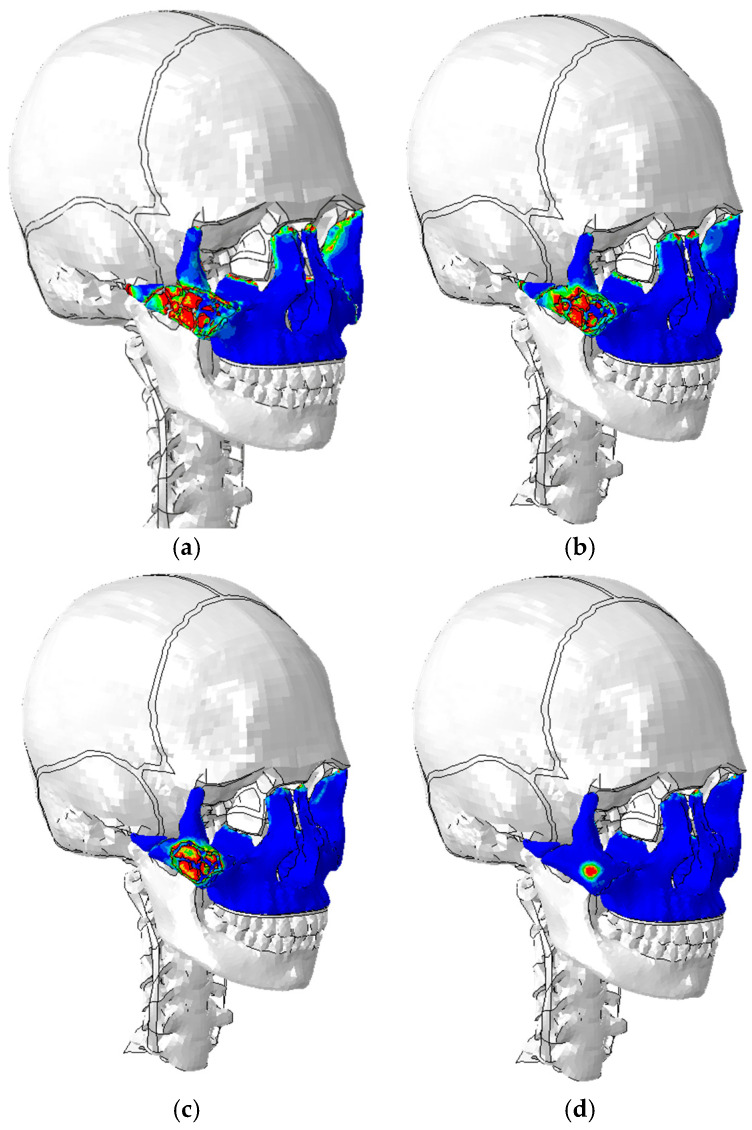
Lines of rupture and initiation of zygomatic bone damage. The entire middle third of the face is highlighted in blue. The red field indicates the initiation of bone damage: (**a**) impact at 20 km/h; (**b**) impact at 15 km/h; (**c**) impact at 10 km/h; (**d**) impact at 5 km/h.

The fracture lines for each of the study configurations are shown in Figure 11.

**Figure 12:**
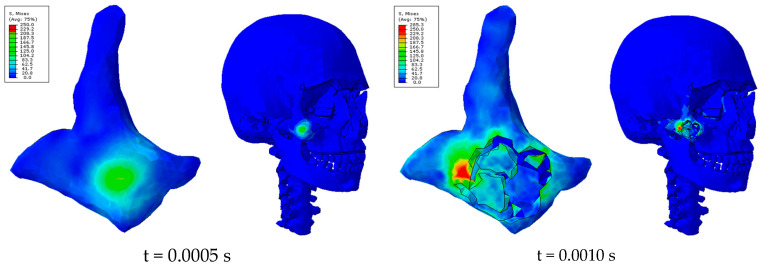

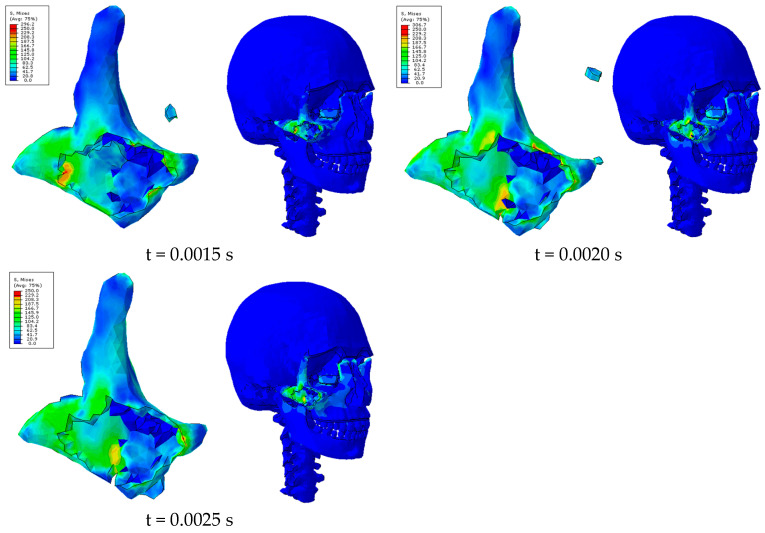
Von Mises stresses [N/mm^2^] zygomatic bone. Impact 15 km/h.

The results presented are based on showing the rupture lines as a function of impactor velocity. To better demonstrate the behavior of the damage criterion used in this study, the values of VonMises stresses obtained in the zygomatic bone for impacts of 15 km/h are shown below (Figure 12).

According to the stress–strain curves in Figure 1, the maximum scale was 250 N/mm^2^, and the maximum strain rate was 1000 (mm/mm)/s.

It is important to note that stress values obtained in some model elements above 250 N/mm^2^ are only derived from the extrapolation and averaging of stresses from the results at the nodes from the results at the integration points and for the graphical representation with continuous stress fields.

It should also be noted that the high stresses obtained, above 80–100 MPa, in the areas of the junction between the zygomatic and maxilla, the zygomatic with supraorbital foramen of the frontal bone, the ethmoid bone, and the frontal process of the maxilla could lead to possible fractures.

## 4. Discussion

The finite-element model used in this study stands out for its complexity and level of detail, representing a significant advance in the simulation of bone fractures, especially in the zygomatic bone. The FEM is a powerful tool for simulating mechanical and structural phenomena in biological tissues. It has been widely used in biomechanical research because it can predict how structures respond to external forces.

In this case, the FEM model includes not only the zygomatic bone but also the mandible, cervical spine, and cervical vertebrae up to C7, encompassing almost the entire stomatognathic apparatus, elements that are essential to simulate force dispersion more realistically. This is crucial because adjacent structures influence how forces impact the zygomatic bone and how it responds. This approach contrasts with previous studies [5,14], where the zygomatic bone was treated as a rigid structure fused to the skull, which simplified interactions but limited accuracy in simulating complex fractures. This model, therefore, represents an evolution concerning the previously mentioned linear models [5,14] since the use of linear materials only allows the evaluation of the stress state before the fracture, not allowing for the study of fracture propagation once the first damage occurs at the bone level.

The model was enhanced using accurate CBCT scan data to obtain the geometry of the zygomatic and maxillary bones, achieving high geometric accuracy. The use of second-order C3D10 tetrahedra to model bones and muscles and first-order C3D8 hexahedral elements for the skull provides adequate resolution to capture the complex interaction between bones and soft tissues. In addition, 1.68 million elements were included in the model, reflecting the high computational complexity involved. In contrast to the models used in previous studies, such as the Huempfner-Hierl et al. (2015) article that uses a total number of 736,934 elements, all of which are second-order tetrahedral, or in contrast to other finite-element studies where the model includes material properties such as Young’s modulus, but the article does not specify the exact number of elements used in the analysis nor their types [5]. In addition, the C3D10 elements, being of second order, offer greater accuracy in the representation of deformation and stress. In the case of the study on composite joints by Blier et al. (2024) [17], the use of C3D8R elements can be justified by the relatively regular geometry of the joints and the focus on strength optimization. C3D8R elements, with their reduced integration point, are computationally less expensive than C3D10 elements, which can be advantageous in optimizations requiring multiple iterations. However, they are more prone to hourglassing, a type of numerical instability. The study’s authors [17] do not mention whether they took steps to mitigate this problem. This approach models the forces accurately, but simplifications still affect the resemblance to reality.

One of the challenges in FEM studies is balancing accuracy with computational feasibility. Although modeled materials (such as cortical and trabecular bone) are based on objective parameters, there are still simplifications, such as approximating bone and tissue properties to homogeneous and continuous behavior. Bones have complex and heterogeneous microstructures that the model cannot faithfully represent. The composition, structure, and mechanical properties of bone vary with direction and location, making it an anisotropic and heterogeneous material [18]. Modeling this heterogeneity is crucial, as it directly influences the mechanical behavior of bone. For example, the spatial distribution of bone mineralization affects the apparent elastic moduli in finite-element models. If this variation in mineralization is not considered, elastic moduli can be significantly overestimated [19].

Several approaches are available to model bone microstructure, including finite-element models that incorporate the geometry, size, volume fraction, and spatial distribution of the different phases of bone, as described in our finite-element study and that of Mischinski and Ural (2013) [6]. Network models describing bone microstructure and its dynamics have also been developed, such as BoneNET, which allows for quantitative assessment of bone mineral density and remodeling dynamics [20].

Accurately capturing these microstructures often requires very fine meshes and complex constitutive models, which can significantly increase computational cost. In addition, accurate data on material properties at the microstructural level can be limited.

Although the skin is not explicitly included in the model, the effect of the skin is simulated using an exponential penetration contact law with a clearance of 1.5 mm, representing the skin’s average thickness in the zygomatic bone area. This delays the onset of the maximum impact force but does not denote substantial changes in the final results. The model represents the same behavior as total contact forces, causing only a temporary delay in reaching the maximum force. These results agree with those reported by Huempfner-Hierl et al. (2015) [14]. However, a redistribution of the force in the contact zone for low–medium-velocity impacts was appreciated. Such a distribution reduces the contact pressure on the bone, reducing the crush fracture. It is also important to note that the skin has a limited capacity to resist compression before a tearing fracture occurs, which would limit its performance in this type of scenario.

The computational complexity of the model is high due to the level of detail and the multidimensional approach. The fact that the model encompasses not only the zygomatic bone but also other adjacent structures and considers nonlinear dynamics implies the need for high processing power. Abaqus Explicit 2023 software, a program recognized for its ability to handle complex biomechanical simulations, was used to perform finite-element calculations. The use of explicit simulation techniques for the impacts (with a minimum time step of 1 × 10^−7^ s) was necessary to hold the highly dynamic nature of the impact events.

One of the significant challenges in FEM simulations is the tuning of the dynamic model. In this study, the model was adjusted and validated by comparing the vibration modes with previous experiments, which allowed accurate calibration to ensure that the simulations reflected the bending and twisting motions of the skull. Despite this, simulation times were extensive, and a mass scaling technique was required to reduce the computational time without compromising the accuracy of the results. This technique consists of slightly increasing the mass of some model elements (in this case, less than 1% of the total mass) to perform calculations in more reasonable times.

One of the most relevant results of the study is the relationship between impact velocity and the type of fracture in the zygomatic bone. For speeds of 20 km/h and 15 km/h, bone crush fractures were observed at impact forces of 400 kg and 300 kg, respectively. These are forces high enough to severely compromise the zygomatic bone structure, implying that, in clinical scenarios such as traffic accidents or high-contact sports, this type of impact would result in severe fractures.

At lower velocities, such as 10 km/h, a mixed-fracture pattern between crushing and bending of the bone and larger bone fragments was observed, suggesting that the bone still has some bending capacity before it fractures ultimately. The maximum contact force for the lowest impact (5 km/h) was approximately 150 kg, which was insufficient to cause a complete fracture; only local damage was detected in the impact zone. These results are essential in assessing the risk of fractures in different scenarios and could help improve injury prevention strategies and face shield design.

Another important aspect is the distribution of contact forces. At impact velocities of 15 km/h, maximum stresses were reached at the junction between the zygomatic bone and adjacent structures, such as the maxilla, the supraorbital foramen of the frontal bone, and the frontal process of the maxilla. Von Mises stresses exceeded 80–100 MPa in these areas, suggesting that they are critical for fracture initiation. The resulting fracture lines varied with impact velocity, extending through the zygomatic arch and other parts of the facial structure, following patterns consistent with clinical observations of zygomatic fractures. One of the main limitations of our study is the lack of a precise quantitative assessment of the evolution of fissures in the zygomatic bone. Although it is possible to assess the amount of crushed bone, the evolution of the fissures cannot be accurately determined with current methods. This aspect is crucial for a more complete understanding of fracture mechanisms and should be addressed in future studies by implementing more advanced techniques for crack quantification and visualization.

The results obtained in this study have direct implications for maxillofacial surgery and the development of zygomatic implants. Predicting how bones react to external forces is critical for designing safer and more effective medical devices. In addition, the findings can be applied to improve protective measures in contact sports and other contexts where the risk of facial trauma is high.

The model could be extended to include the interaction of zygomatic implants with bone and explore individual variability in bone density and morphology, which could provide more personalized and relevant simulations for specific patients.

## 5. Conclusions

This study demonstrates the finite-element method’s potential to simulate the zygomatic bone’s behavior upon impact, achieving accurate and clinically relevant results.

Through a detailed and realistic approach that includes not only the zygomatic bone but also nearby structures such as the mandible, the cervical spine, and, in general, all the structures of the stomatognathic apparatus, more complete simulations have been achieved that can be applied in various areas of maxillofacial surgery and trauma prevention.

The high computational complexity of the models reflects the technical challenge of accurately simulating biomechanical interactions. Still, the results are valuable for understanding bone fractures and their clinical implications.

## Figures and Tables

**Table 1:** Initial data for type II bone were used in the numerical models according to Misch’s classification.

	Young’s Modulus (GPa)	Poisson’s Ratio	Density (g/cm)^3^
Cancellous bone	5.5	0.3	2.12
Cortical bone	13.7	0.3	2.12

**Table 2:** Damage initiation parameters with ductile rupture criterion.

$\mathrm{Equivalent}\;\mathrm{Rupture}\;\mathrm{Deformation}\;\mathrm{at}\;\mathrm{the}\;\mathrm{Onset}\;\mathrm{of}\;\mathrm{Damage}\;\varepsilon_{t}^{pl}$	Invariant −p/q	$\mathrm{Strain}\;\mathrm{Rate}\;\dot{\varepsilon }$
0.02	0.33	0
0.02	0.33	0.01
0.0175	0.33	0.1
0.0149	0.33	1.0
0.0123	0.33	10.
0.0072	0.33	100.
0.0024	0.33	1000.

## Data Availability

This manuscript contains all necessary data regarding the research. If any reader has any questions, we recommend that they contact the corresponding authors.
